# The impact of tinnitus on speech perception in noise: a systematic review and meta-analysis

**DOI:** 10.1007/s00405-024-08844-1

**Published:** 2024-07-26

**Authors:** Sanjana Madhukesh, Hari Prakash Palaniswamy, Kanaka Ganapathy, Bellur Rajashekhar, Kavassery Venkateswaran Nisha

**Affiliations:** 1 Department of Speech and Hearing, Manipal College of Health Professions (MCHP), Manipal Academy of Higher Education (MAHE), Manipal, Karnataka India; 2 Department of Audiology, All India Institute of Speech and Hearing, Mysuru, Karnataka India

**Keywords:** Tinnitus, Auditory processing, Speech perception, Cognitive function, Hearing loss

## Abstract

**Purpose:**

Tinnitus is a condition that causes people to hear sounds without an external source. One significant issue arising from this condition is the difficulty in communicating, especially in the presence of noisy backgrounds. The process of understanding speech in challenging situations requires both cognitive and auditory abilities. Since tinnitus presents unique challenges, it is important to investigate how it affects speech perception in noise.

**Method:**

In this review, 32 articles were investigated to determine the effect of tinnitus on the effect of speech in noise perception performance. Based on the meta-analysis performed using a random-effects model, meta-regression was used to explore the moderating effects of age and hearing acuity.

**Results:**

A total of 32 studies were reviewed, and the results of the meta-analysis revealed that tinnitus significantly impacts speech in terms of noise perception performance. Additionally, the regression analysis revealed that age and hearing acuity are not significant predictors of speech in noise perception.

**Conclusion:**

Our findings suggest that tinnitus affects speech perception in noisy environments due to cognitive impairments and central auditory processing deficits. Hearing loss and aging also contribute to reduced speech in noise performance. Interventions and further research are necessary to address individual challenges associated with continuous subjective tinnitus.

## Introduction

Tinnitus is a common hearing condition affecting 14% of individuals worldwide [1]. It is the conscious awareness of composite or tonal stimuli for no known corresponding external auditory source [2]. Tinnitus severity varies widely from person to person, with some individuals experiencing mild annoyance and others experiencing severe impacts on their daily lives. Beyond its intrusive auditory nature, tinnitus can have various consequences for an individual’s life, including poor emotional well-being, poor sleep quality, and the ability to communicate effectively in noisy environments [3–5]. Various factors, including noise exposure, ear infections, age-related hearing impairment, and ototoxic medication-related side effects, can cause tinnitus [6].

Speech perception in noise refers to an individual’s ability to understand spoken language in background noise. This complex task demands an individual’s auditory and cognitive abilities [7–9]. Individuals with tinnitus often experience speech perception deficits despite normal hearing [10, 11]. In contrast, the literature suggests that speech in noise perception is unaffected in individuals with tinnitus and normal hearing [12]. The mixed results of the relationship between tinnitus and speech perception in noise are complex and based on multiple factors. Several mechanisms have been proposed to explain speech processing deficits in individuals with tinnitus. They are reportedly related to peripheral hearing impairment and central auditory processing dysfunction, the influence of cognitive control mechanisms [13], and attention-based networks [14]. These proposed mechanisms are influenced by various factors, such as the degree of hearing loss, tinnitus severity, age, sex, and hearing aid use [12, 15–17].

Despite the increasing interest in exploring the effect of tinnitus in various central auditory processing tasks, particularly speech in noise, the existing studies differ significantly in terms of the target stimulus type or masker. The preferred masker stimuli are babble speech, with the kind of babble varying across studies from a single talker [10] to an 8-talker [18, 19]. In terms of target stimuli, studies have used a variety of speech tokens ranging from consonant vowels (CVs) [20, 21] to sentences [4, 12, 22].

The literature suggests that tinnitus may interfere with auditory processing, leading to difficulties in speech perception, especially in noisy environments [23–25], but the underlying mechanism remains unclear. The literature suggests complex interactions between tinnitus and other factors, such as age, cognition, and hearing status [26, 27]. Tinnitus rarely presents solitarily; rather, it often coexists with hearing loss. Tinnitus often occurs alongside hearing loss, raising questions about whether tinnitus alone can affect speech perception, whether these effects are combined, or whether hearing loss might mask the effects of tinnitus [23, 28]. Additionally, age-related changes in auditory processing and cognitive function may further contribute to poor speech perception in tinnitus patients [15, 29, 30]. In addition, the variation in material and quantification methods also varies across studies [31, 32]. In the literature, speech in noise has been studied using materials ranging from phonemes to sentences. The cognitive resources required to process sentences or words differ significantly [33]. Similarly, the type of background noise can differentially affect speech in noise perception. It has been demonstrated that different types of maskers can produce varying levels of interference, with speech maskers leading to poorer scores than steady noise [34].

We hypothesize that tinnitus negatively impacts speech in noise perception and that this effect is moderated by various factors, such as type of hearing acuity, age, type of target, and masker. The current systematic review and meta-analysis aimed to comprehensively examine the impact of tinnitus on speech-in noise perception. Through meta-regression, factors such as hearing acuity, age, and target-masker type were quantified to explore the influence of these moderators on the relationship between tinnitus and speech perception in noise performance. Thus, this study offers a thorough understanding of the effects of tinnitus on speech perception in noise.

## Methods

This study aimed to systematically review peer-reviewed, published studies employing speech-in-noise perception tests in individuals with tinnitus. The study was preregistered and approved by PROSPERO (ID: CRD42022350779). The current systematic review adhered to the PRISMA guidelines [35].

### Eligibility criteria

The inclusion criteria were based on the Cochrane guidelines for conducting systematic reviews [36]. The review consists of studies consisting of individuals with continuous tinnitus with or without hearing loss. The individuals were adults aged > 18 years, irrespective of sex. The review included studies assessing various objective measures of speech in noise perception tests, irrespective of the type of stimuli and the type of interpretation. All the studies were original research articles, such as randomized controlled trials, observational studies, and cohort studies. Studies comprising a control group, an experimental group or an experimental group alone were included in the review. Other study designs, such as case reports, case series and animal studies and reviews, were excluded. Furthermore, if the studies had intervention as the main objective, only the prespeech in noise perception scores were considered for review. The criteria are depicted in detail in Table 1.

**Table 1:** Eligibility criteria of the potential studies included

PECOT	Inclusion criteria	Exclusion criteria
Population (P)	Adults (> 18 years) irrespective of gender were included for the review. Individuals with continuous tinnitus with and without hearing loss were included in the review process Preintervention scores (hearing aids and cochlear implants) were included	Adults with intermittent and pulsating tinnitus Animal studies Individuals with hearing aids and cochlear implants
Exposure (E)	Studies assessing different tests of speech in noise perception Studies that have used speech perception measures in noise tests, irrespective of interpretation of the tests	Studies that have assessed speech perception in noise postintervention, such as hearing aids and cochlear implants
Comparison (C)	Individuals with normal hearing thresholds Individuals without tinnitus complaints	NA
Outcome (O)	Objective speech in noise tests, irrespective of the type of stimuli, noise and methodological variation	Outcome measures specified other than the inclusion criteria: subjective measures
Type of the study	Original research studies, including randomized controlled trials, observational studies, cohort studies, and case‒control studies	Other study designs, study protocols, study reports, case series reports, systematic reviews and meta-analysis

*NA* not applicable

### Literature search strategy and information sources

**Fig. 1:**
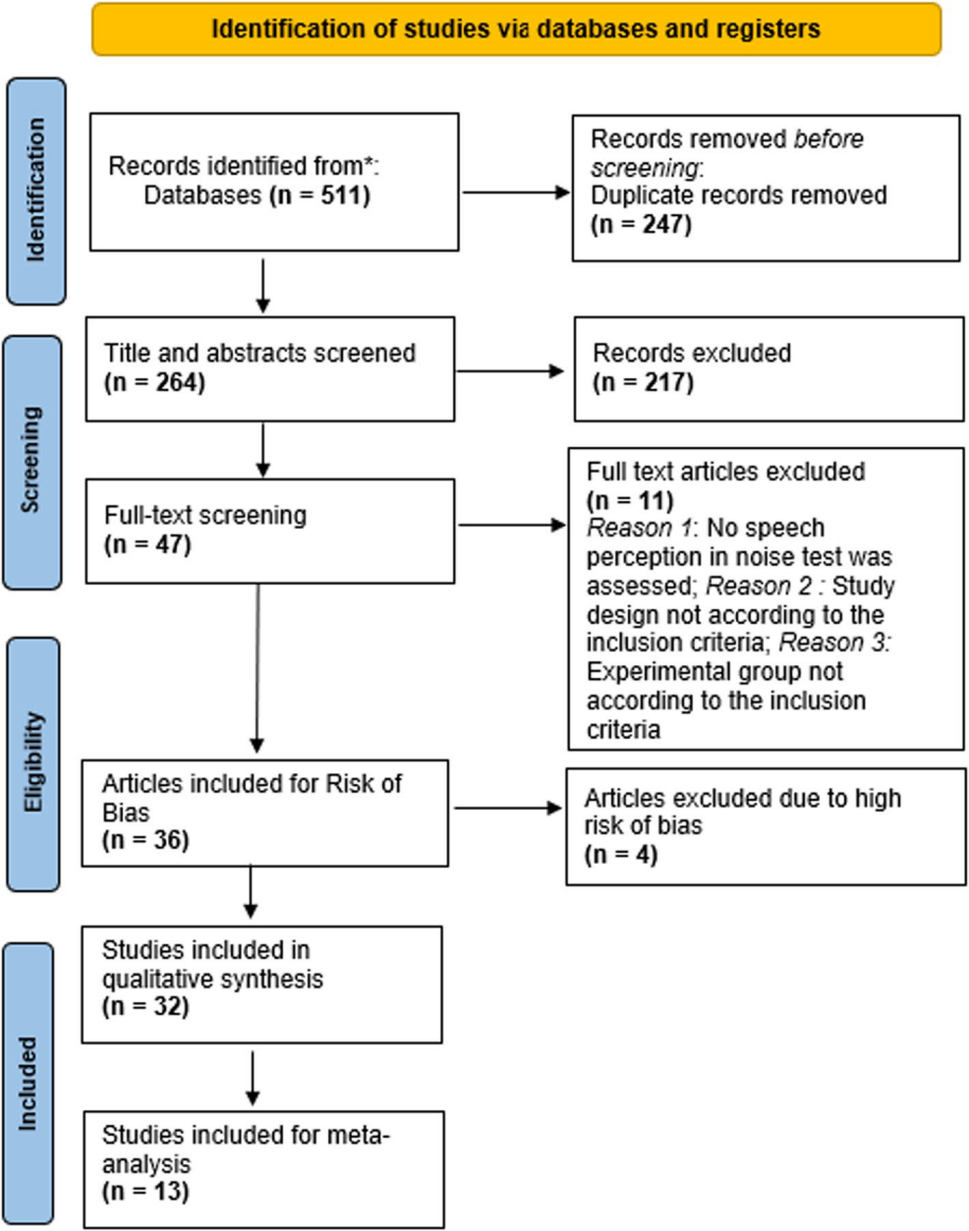
The PRISMA flowchart visually summarizes the systematic review process

Based on the databases, using the keywords "tinnitus" along with "speech in noise perception," a search string was constructed using appropriate Boolean operators (Keywords: (tinnitus [MeSH]) AND ("speech in noise" OR "speech perception in noise" OR "sentence in noise" OR "signal-to-noise ratio" OR "SNR" OR "signal in noise" OR "word in noise" OR "speech recognition in noise"). The keywords generated articles from five databases: PubMed, Scopus, Web of Science, CINAHL, and ProQuest. There were no restrictions on the language of the search strategy. The search was predominantly run through the title and abstract of all articles until January 10, 2023. Using Covidence software, 264 articles were retained after removing duplicates (n = 247) (Fig. 1).

### Screening of articles

Two independent reviewers screened the articles based on title and abstract (Reviewer 1, SM and Reviewer 2, NK). Reviewer 3, KG, resolved the conflicts from this initial screening. Initially, 511 articles were identified through a primary database search. After the removal of duplicates, 264 studies remained for the initial screening. Subsequently, a thorough examination of the titles and abstracts led to the inclusion of 47 studies for full-text analysis. After an additional in-depth review, 32 studies were deemed suitable for inclusion in the systematic review, while articles with a high risk of bias were excluded. For a detailed illustration of the process and the exclusion criteria, refer to Fig. 1, which presents the PRISMA flowchart.

### Extraction of data

Data extraction was independently performed by two reviewers (SM and NK), and a data check was performed by a third reviewer (KG). The extracted data included the following: type of study design, place of study, age and gender of participants, mean and standard deviation (SD) values of the speech in noise perception test as the primary outcome variable and other data, such as hearing and tinnitus characteristics. The unreported means and SDs were requested from the corresponding authors or extracted from the figures using the WebPlot digitizer online tool [37].

### Risk of bias (RoB) analysis

Quality assessment of the 36 eligible studies was carried out by two independent reviewers (BR and SM), and conflicts were resolved by HP. The Critical Appraisal Skilled Programme (CASP) [38], which is based on case‒control studies, was used for risk analysis. The studies were evaluated based on whether they utilized a thorough aim of the study, methodology, the credibility of the findings, and their relevance from a clinical perspective. Questions such as “did the study address a clearly focused issue?”, “Did the authors use appropriate method to answer the question?”, “Were the controls selected in an appropriate way?”, and many more were used to qualitatively assess the studies. The last column consisted of the overall quality of the studies based on the various domains, and this was considered the final result. Based on these factors, the reviewers were instructed to rate the overall risk as either "High", "Average", or "Low". A third reviewer resolved conflicts when the two results were opposite to each other, and this was considered the final qualitative assessment. Based on the overall input from the reviewers, four articles were excluded (Table 2).

**Table 2:**
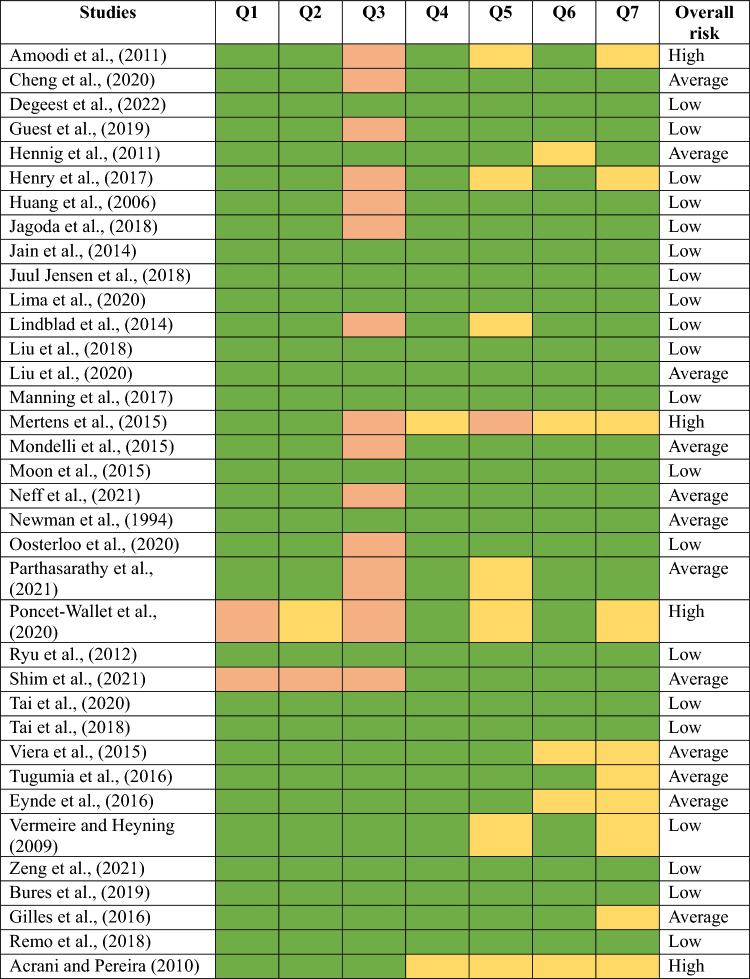
Results of risk of bias (RoB) analysis across studies using the Critical Appraisal Skills Programme (CASP) Checklist

Q1 = Did the study address a focused issue? Q2 = Did the authors use an appropriate method to answer their question? Q3 = Were the controls selected in an appropriate way?; Q4 = Was the exposure accurately measured to minimum bias?; Q5 = Have the authors taken account of the potential confounding factors in the design and/or their analysis?; Q6 = How precise was the estimation of effect? and Q7 = Can the results be applied to the local population? The responses to the questions were categorized as green for yes, yellow for unsure, and orange for no. Each study was rated as high, average, or low risk

### Data synthesis

The extracted data were synthesized into a narrative form under various categories, including place of the study, age and gender of the participants, characteristics of tinnitus, hearing acuity of the participants, tinnitus assessment, tools used for evaluating the severity and impact of tinnitus and interpretation of the speech in noise perception scores.

### Meta-analysis and meta-regression

Comprehensive meta-analysis software (version 4) [39] was used to analyze the influence of tinnitus on speech perception in noise scores, combining the means and SDs of the speech in noise perception scores and assessing heterogeneity. The analysis used the random-effects model to allow the results to be generalized to other studies [40]. Continuous variables were estimated using differences in means with 95% confidence intervals (CIs). The absolute heterogeneity level among the effect estimates was assessed by calculating the between-studies SD and 95% prediction interval (PI). *I*^2^ values of < 25%, 25 to 50%, and > 50% represented low, moderate, and high heterogeneity, respectively. Furthermore, to investigate the potential role of moderators such as hearing acuity, age and target type on the influence of speech perception in noise scores among the tinnitus population, a subgroup meta-regression was performed.

## Results

### Narrative synthesis

**Fig. 2:**
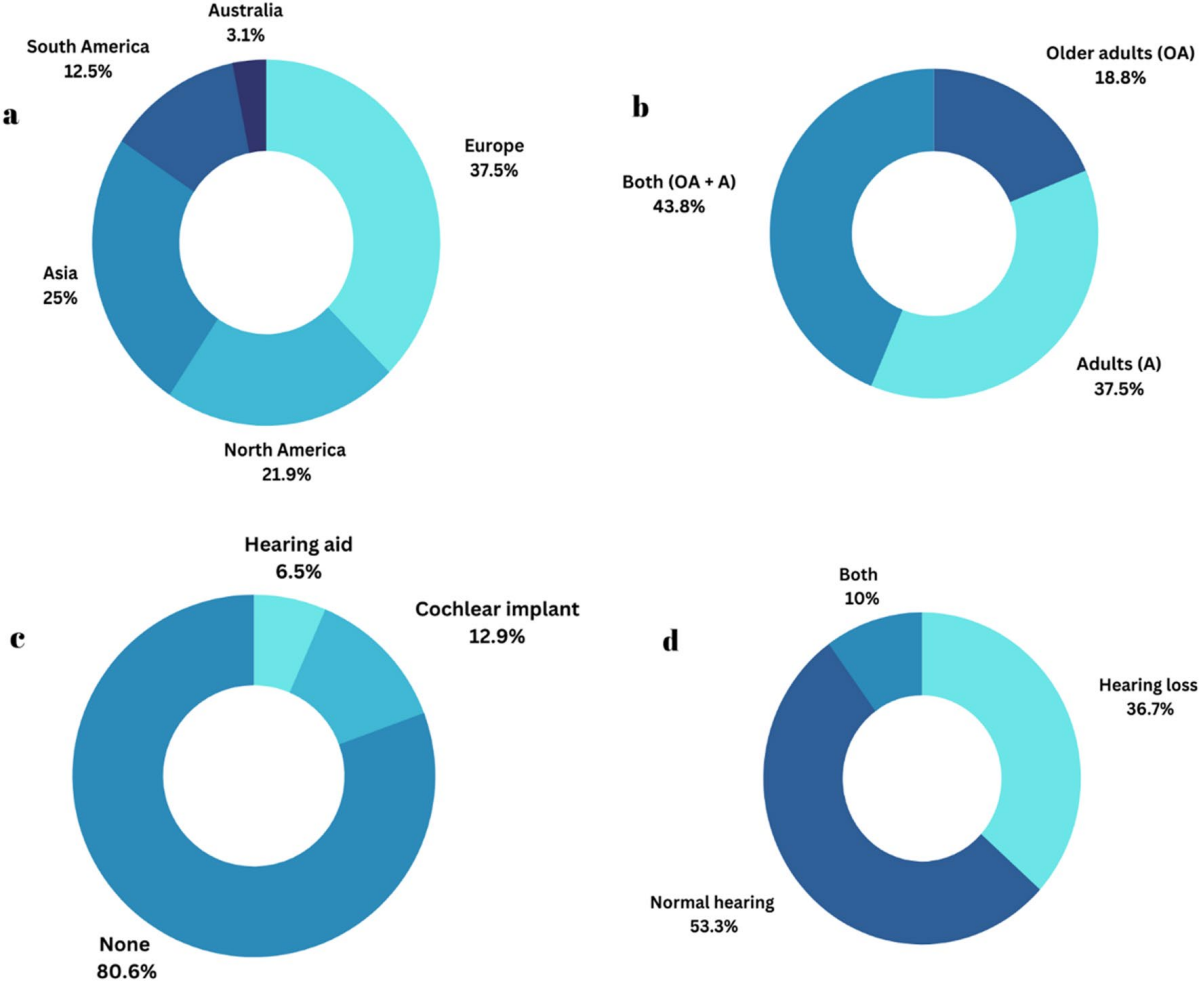
The total number of studies based on **a** place of study, **b** age group, **c** type of amplification device, and **d** hearing status

Overall, the current systematic review included 32 published studies for qualitative analysis. The overall summary of the reviews, such as demographic information, is presented in Fig. 2.

#### Place of study

Twelve studies were conducted in various European countries, including the United Kingdom, Belgium, Netherlands, France, Denmark, Sweden, and Austria. In addition, seven studies were carried out in the United States of America; eight in Asian countries such as Taiwan, Korea, India, and China; four in South America; and one in Australia. Figure 2a depicts an overview of the different places where the study was conducted.

#### Age and sex

Fourteen studies included participants with tinnitus ranging in age from 18 to 75 years. However, 12 studies included only younger adults (from 18 to 55 years), and six studies included only older adults (from 55 to 75 years). Two studies classified the participants into young and older groups with tinnitus [12, 41]. The proportion of this classification is depicted in Fig. 2b.

#### Hearing acuity

In most of the studies [number of studies (N) = 17], routine pure-tone audiometry was used, while in a few studies, high-frequency pure-tone audiometry was used (N = 3). The review included the mean threshold of the control group, which was 18.5 dB HL, and that of the tinnitus group, which was 31.6 dB HL. The mean thresholds were calculated as three-tone averages of 500 Hz, 1000 Hz and 2000 Hz bilaterally, except for the study by Tai and Hussain [61], which included right- and left-ear-specific thresholds. Out of the 32 studies, 16 reported a pure tone average of participants less than 25 dB HL, and four studies categorized the hearing thresholds as mild severity. Fifteen studies included participants with more severe hearing loss (ranging from 40 HL to 108.51 dB HL). Figure 2c, d provide information on the total number of studies conducted on hearing status and the type of amplification devices used.

#### Tinnitus characteristics

As per the inclusion criteria of the current review, all the studies included only participants with continuous tinnitus. The mean tinnitus duration reported in ten studies was 7.04 years (ranging from 1 month to more than 10 years). Fifteen studies reported on individuals with more than 6 months of tinnitus. Three studies included participants with more than 3 months of tinnitus [10, 42, 43]. One study by Van Eynde et al. [44] included five participants with less than 1 month of tinnitus. Eight of the 32 studies included pitch-related information, and eight studies included loudness-based information (Tables 3, 4). The pitch and loudness measures were either included descriptively (tonal, noise-like pitch), the mean of the pitch matched, or the individual pitch of the participants. Tinnitus in both ears was reported in 12 studies, and unilateral tinnitus was reported in 13 studies, of which ten studies reported the laterality of tinnitus. Studies have also classified tinnitus as central (midline or global perception) and unknown laterality [15, 44–46]. The tinnitus characteristics are depicted as two separate tabular columns based on the inclusion of the tinnitus group + control group (Table 3) and the tinnitus group alone (Table 4).

**Table 3:** Tinnitus characteristics of the studies including both tinnitus and control group

Study (no. of tinnitus participants)	Duration of tinnitus (months/years)	Laterality (n)	Pitch (Hz)	Loudness (dB HL)	Scales used
Hennig et al., 2011 [4] (n = 19)	NR	NR	NR	NR	THI
Huang et al., 2006 [16] (n = 20)	Mean: 65.79 months	Rt: 2; Lt: 3; B/L:15	NR	NR	THI, TLS, and VAS
Ryu et al., 2012 [24] (n = 20)	Mean: 16.2 months	Rt: 14; Lt: 6	< 500 Hz: 4 500–4 kHz: 6 > 4 kHz: 6 Undetermined: 4	NR	THI
Newman et al., 1994 [55] (n = 23)	Mean: 10.7 years	Rt: 8; Lt: 15	Mean pitch: 5.04 kHz	Mean: 50 dBHL	ARS, THQ
Liu et al., 2018 [80] (n = 14)	NR	NR	NR	NR	THI and VAS
Moon et al., 2015 [58] (n = 30)	Group 1: 10.22 months Group 2: 60.67 months Group 3: 51.25 months	U/L: 21; B/L: 9	*Mean pitch* Group 1: 4 k Hz Group 2: 5.27 kHz Group 3: 6.38 kHz	NR	THI, TAS, and VAS
Jain and Sahoo, 2014 [18] (n = 20)	NR	NR	NR	NR	THI
Guest et al., 2019 [10] (n = 67)	3 months and above	NR	NR	NR	TFI
Degeest et al., 2022 [43] (n = 13)	3 months above	B/L: 10 U/L: 3 (Rt: 2 and Lt: 1)	Tonal: 92.3% and noise-like: 7.7%	NR	THI and TFI
Tai and Husain, 2018 [61] (n = 14)	Mean: 17.71 years	B/L and louder in Rt: 1 B/L and louder in Lt: 2 B/L only: 11	NR	NR	THI
Gilles et al., 2016 [45] (n = 19)	3 months and above; Mean: 2 years	B/L: 16; U/L: 2 and Central: 1	Pure tones: 13 Noise: 6	NR	TQ and VAS-L
Bures et al., 2019 [15] (n = 25)	NR	Central: 13; Lt: 7 and Rt: 5	NR	NR	THI
Oosterloo et al., 2020 [23] (n = 877)	Options provided: More than once a week but not daily or daily	NR	NR	NR	THI-simplified version
Lima et al., 2020 [20] (n = 15)	6 months and above; Mean: 5.04 years	B/L: 3, Rt: 4; Lt: 5; Rt > Lt: 3	Whistling: 9 Wheezing: 4 Pulsatile: 2 Mean of Rt and Lt: 3000 Hz	Mean: 13 dB SL in both ears	THI and VAS
Liu et al., 2020 [56] (n = 10)	Mean: 1.84 years	B/L: 6; Rt: 1; Lt: 3	NR	NR	THI and VAS
Tai and Husain, 2020 [17] (n = 34)	6 months and above Tin-NH: 7.58 years Tin-HL: 13.72 years	B/L: 26; U/L: 4; Head: 4	Tin-NH: 3808.82 Hz Tin-HL: 3455.88 Hz	NR	TFI
Zeng et al., 2020 [12] (n = 45)	6 months and above	NR	Mean: 4 kHz	Mean: 10 dB SL (Young: 11.4 dBSL and Old: 9.1 dB SL)	TFI
Cheng et al., 2020 [42] (n = 18)	3 months and above	B/L	NR	NR	NR
Jensen et al., 2018 [19] (n = 16)	6 months and above	NR	NR	NR	THI, Tinnitus thermometer

According to the study by Moon et al. [58], the three groups were classified as follows: group 1, tinnitus with normal hearing thresholds; group 2, unilateral tinnitus with hearing loss; and group 3, bilateral tinnitus with hearing loss

*NR* not reported, *Rt* right, *Lt* left, *B/L* bilateral, *U/L* unilateral, *NH* normal hearing, HL hearing loss, *Tin* tinnitus, *THI* tinnitus handicap inventory, *TFI* tinnitus functional index, *VAS-L* visual analog scale for loudness, *TQ* tinnitus questionnaire, *TLS* tinnitus loudness scaling, *ARS* annoyance rating scale, *THQ* tinnitus handicap questionnaire

**Table 4:** Tinnitus characteristics of studies comprising the tinnitus group alone

Study (no. of tinnitus participants)	Duration of tinnitus (months/years)	Laterality	Pitch	Loudness	Scales used	Study remarks
Eynde et al., 2016 [44] (n = 37)	< 1 month: 9; 1 to 12 months: 13; > 12 months: 15	Lt: 3; Rt: 8; B/L: 12; Unknown: 5	NR	NR	TQ and THI	Correlated with tinnitus characteristics
Shim et al., 2021 [41] (n = 13)	Mean: 30.08 months	B/L: 5; Lt: 6; Rt: 2	NR	Mean: 3.83 dB SPL	THI, TAS, VAS	Intervention using HA and HA + Tinnitus sound control
Távora-Vieira et al., 2015 [62] (n = 13)	NR	NR	NR	NR	TRQ	CI intervention
Mertens et al., 2015 [53] (n = 22)	Less than 10 years	U/L	NR	NR	NR	CI intervention
Arts et al., 2018 [51] (n = 10)	NR	U/L	NR	NR	NR	CI intervention and auditory training
Vermeire and Van De Heyning, 2009 [54] (n = 20)	Since the time of deafness	U/L	NR	NR	NR	CI intervention
Lindblad et al., 2014 [52] (n = 175) (n = 193 total participants; with 69% with T and HP; 22% T alone)	NR	NR	NR	Median levels: Lt: 40 dB HL Rt: 38 dB HL	Case history questionnaire with tinnitus-related questions	Subgroup of tinnitus and SRT
Henry et al., 2017 [60] (n = 55)	NR	NR	NR	NR	TFI	Efficacy of HA, HA + SG, and deep fit HA
Jagoda et al., 2018 [46] (n = 30)	Mean: 12 years	B/L: 17; Rt: 6 and Lt: 7	NR	NR	THI and TQ	Neurophysiological study
Tugmia et al., 2016 [81] (n = 12)	NR	B/L: 9; Rt: 2; Lt: 2	CG: 7675 Hz SG: 7606 Hz	CG: 19 dB SL SG: 7.27 dB SL	THI	Auditory training
Mondelli et al., 2021 [22] (n = 35)	6 months and above	B/L	Mean: Group 1: 3.30 kHz Group 2: 4.37 kHz Group 3: 3.72 kHz Group 4: 5.44 kHz	Mean: 7.03 dB SPL	THI and VAS	HA and sound generators
Neff et al., 2021 [57] (n = 146)	3 months and above; Mean: 180 months	NR	NR	NR	TQ	SRT with cognitive measures
Parthasarathy and Shetty, 2021 [59] (n = 20)	NR	U/L: 18; B/L: 2	Tonal; Mean: 2175 Hz	Mean: 64.75 dB HL	THI	Different types of amplification strategies

In the study by Mondelli et al. [22], the groups were classified based on the types of management provided, i.e., Group 1: White noise, Group 2: Pink noise, Group 3: Speech noise, and Group 4: High tone

*NR* no report, *Rt* right, *Lt* left, *B/L* bilateral, *U/L* unilateral, *NH* normal hearing, *HL* hearing loss, *Tin* tinnitus, *THI* Tinnitus Handicap Inventory, *TFI* Tinnitus Functional Index, *VAS-L* Visual Analog Scale for Loudness, *TQ* Tinnitus Questionnaire, *TAS* Tinnitus Annoyance Scale, *ARS* Annoyance Rating Scale, *THQ* Tinnitus Handicap Questionnaire, *TRQ* Tinnitus Reaction Questionnaire, *SRT* speech recognition threshold, *CG* control group, *SG* study group, *HA* hearing aid, *SG* sound generators, *CI* cochlear implant

#### Tools used for evaluating the severity and impact of tinnitus

The most commonly used questionnaires/scales were the Tinnitus Handicap Inventory (THI) [47] (N = 20), followed by the Tinnitus Functional Index (TFI) [48] (N = 5) and the Tinnitus Questionnaire [49] (n = 4). Studies have also used the Tinnitus Reaction Questionnaire (TRQ) [50] (N = 1) to measure tinnitus severity. However, five studies did not report scales or questionnaires to measure tinnitus [42, 51–54]. Apart from the commonly used measures, the loudness or annoyance of tinnitus was measured using the tinnitus loudness scale (TLS) [16] and the annoyance rating scale [55]. The analog scale for loudness and annoyance was used in the following studies Gilles et al. [45]; Huang et al. [16]; Liu et al. [56]; and Mondelli et al. [21].

Of the 32 studies included in the review, 13 included only a tinnitus group and did not include a control group. These studies reported decreased speech-in-noise scores, aligning with the main objective of their research, which was to assess the effectiveness of interventions such as hearing aids, sound generators, cochlear implants, and auditory training. Six studies included intervention studies consisting of pre- and postspeech performance in noise. Similarly, four studies explored the effectiveness of hearing aids with or without sound generators in treating tinnitus. Other studies have explored speech-in-noise performance and correlated the scores with tinnitus characteristics and audiometric thresholds [44, 46, 52, 57].

#### Speech in noise measurement

The studies included a variety of target and masker combinations. Of these, the most commonly used targets were sentence materials (N = 24), followed by words and phonemes/syllables [20, 21, 23, 41, 42, 44, 57, 58] (N = 8). Speech-shaped noise was the most commonly used noise type (n = 10) [4, 16, 24, 51, 53, 59], and multitalker babble noise was most commonly used (n = 11) [15, 18, 19, 46, 55–57, 60–62]. The other types of noise used in speech in noise perception tests are spatial noise, competing signals, and amplitude-modulated noise [10, 12, 20, 45, 52]. A detailed representation of the target and masker characteristics is presented in Table 5.

**Table 5:** Characteristics of various speech-in-noise tests

Study	Test type	Target stimulus	Masker type	Outcome measure
Hennig et al., 2011 [4]	Speech recognition threshold in noise	Phonetically balanced sentences (four to seven words)	Speech-shaped noise	Signal to noise ratio
Huang et al., 2006 [16]	Mandarin Speech Perception in Noise test	Sentences (high and low predictability)	Speech shaped noise	Raw scores
Ryu et al., 2012 [24]	Korean version of the Hearing in Noise Test	Sentences	Speech-spectrum shaped noise	Signal to noise ratio
Newman et al., 1994 [55]	Speech perception in noise test (SPIN)	Sentences (high and low predictability)	Multitalker speech babble	Signal to noise ratio
Liu et al., 2018 [80]	Speech recognition in noise	Sentences	Spatial noise	SRT (dB)
Moon et al., 2015 [58]	Speech recogniton threshold in noise	Spondee words (open-set)	Speech-shaped steady noise	dB SNR
Jain and Sahoo, 2014 [18]	Quick speech in noise test (Kannada)	Sentences	Four-talker speech babble	SNR50
Guest et al., 2019 [10]	Speech in noise test	Coordinate-Response-Measure (CRM) phrases at zero degree azimuth	Two-talker interference	dB SNR
Degeest et al., 2022 [43]	Speech understanding test	Digits	Tonal: 92.3% and noise-like: 7.7%	SNR
Tai and Husain, 2018 [61]	Quick speech in noise test	Sentences	Multitalker babble	SNR loss
Gilles et al., 2016 [45]	Speech in noise	Dutch sentences: Leuven Intelligibility Sentence Test	Amplitude modulated noise	dB SNR
Bures et al., 2019 [15]	Speech in noise	Czech sentences	Babble noise	SNR 50
Oosterloo et al., 2020 [23]	Digits in noise test	Digit triplets	Speech shaped noise	Speech reception threshold in noise
Lima et al., 2020 [20]	Speech in noise test	Monosyllables	Competing message	Raw scores
Liu et al., 2020 [56]	Mandarin Speech Perception test	Sentences	Six-talker babble	SRT
Tai and Husain, 2020 [17]	Quick speech in noise test	Sentences	Four-talker babble	SNR loss
Zeng et al., 2020 [12]	Speech in noise perception test	Sentences	Noise matched to the frequency spectrum of the target speaker	Mean: 10 dB SL (Young: 11.4 dBSL and Old: 9.1 dB SL)
Cheng et al., 2020 [42]	Speech in noise perception test	Spondee words	Speech-shaped noise	SNR 50
Jensen et al., 2018 [19]	Danish version of Hearing in Noise Test	Sentences	Four-talker babble	dB SNR
Eynde et al., 2016 [44]	Speech in noise test: Digit triple test	Digits	Adaptive noise procedure	SRT scores
Shim et al., 2021 [41]	Speech recognition threshold in noise	Spondee words	Speech-shaped steady noise	SRT
Távora-Vieira et al., 2015 [62]	Bamford-Kowal-Bench Speech-in-Noise (BKB-SIN)	Sentences	Multitalker babble	SNR
Mertens et al., 2015 [53]	LIST (Leuven Intelligibility Sentences Test)	Sentences	Speech-weighted noise	dB SNR
Arts et al., 2018 [51]	LIST (Leuven Intelligibility Sentences Test)	Sentences	Speech-weighted stationary noise	dB SNR
Vermeire and Van De Heyning, 2009 [54]	Leuven Intelligibility Sentence Test (LIST)	Sentences	Speech-weighted stationary noise	dB SNR
Lindblad et al., 2014 [52]	Speech recognition threshold in modulated noise	Hagerman's 5-word sentences	Modulating noise between 1 to 5 Hz and similar to modulated speech spectrum	dB threshold
Henry et al., 2017 [60]	Quick speech in noise test	Sentences	Multitalker babble	SNR loss
Jagoda et al., 2018 [46]	Speech in noise test	Sentences	Multitalker babble	dB SNR
Tugmia et al., 2016 [81]	Speech in noise test (as central auditory processing battery)	NR	NR	NR
Mondelli et al., 2021 [22]	Hearing in Noise Test	Sentences	Quiet and frontal noise	dB SPL
Neff et al., 2021 [57]	German speech-in-noise test	Three groups of words with identical recognition difficulty	Speech babble	Hit rate %
Parthasarathy and Shetty, 2021 [59]	Speech in noise test	Standardized (phonetically and phonemically balanced) sentences	Speech-shaped noise	dB

*SNR* signal-to-noise ratio, *dB* decibel (unit), *SPL* sound pressure level, *SRT* speech recognition threshold, *NR* not reported

### Meta analyses

A total of 13 studies were included in the meta-analysis based on the study's primary aim and the data availability.

#### Meta-analysis: speech in noise perception test

**Fig. 3:**
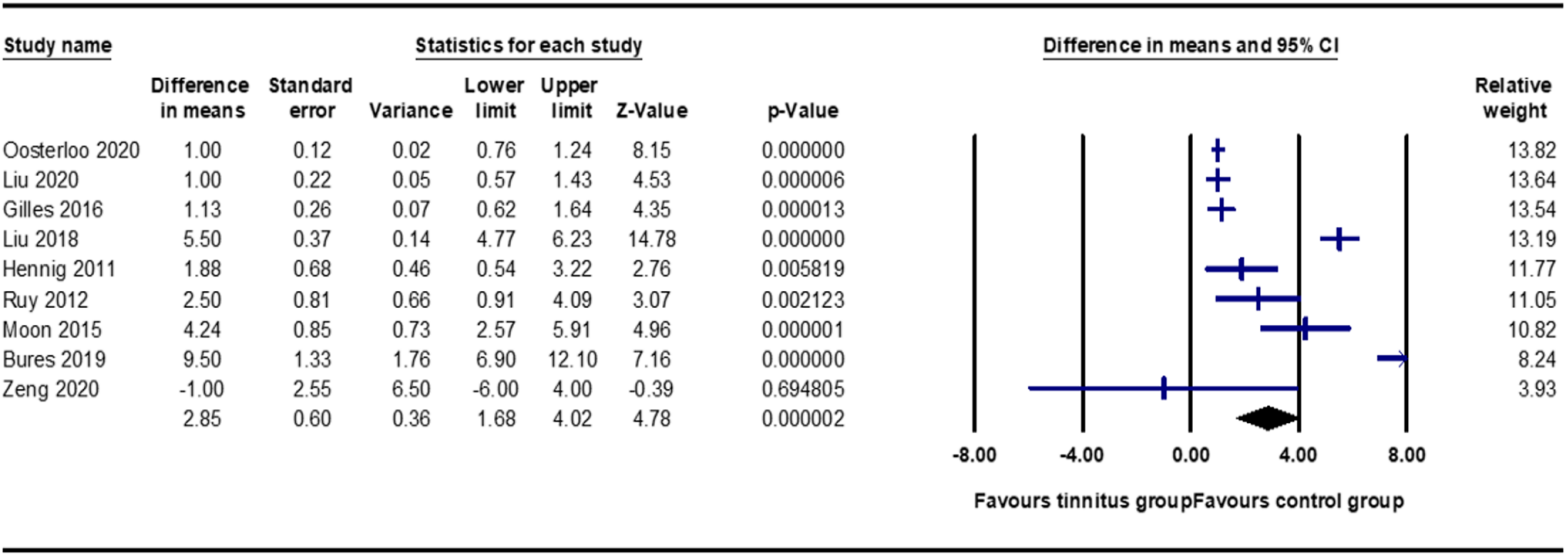
The meta-analysis of speech perception in the noise test was interpreted as the SNR50. A total of eight studies were arranged according to the relative weight and depicted along with the respective forest plots

**Fig. 4:**
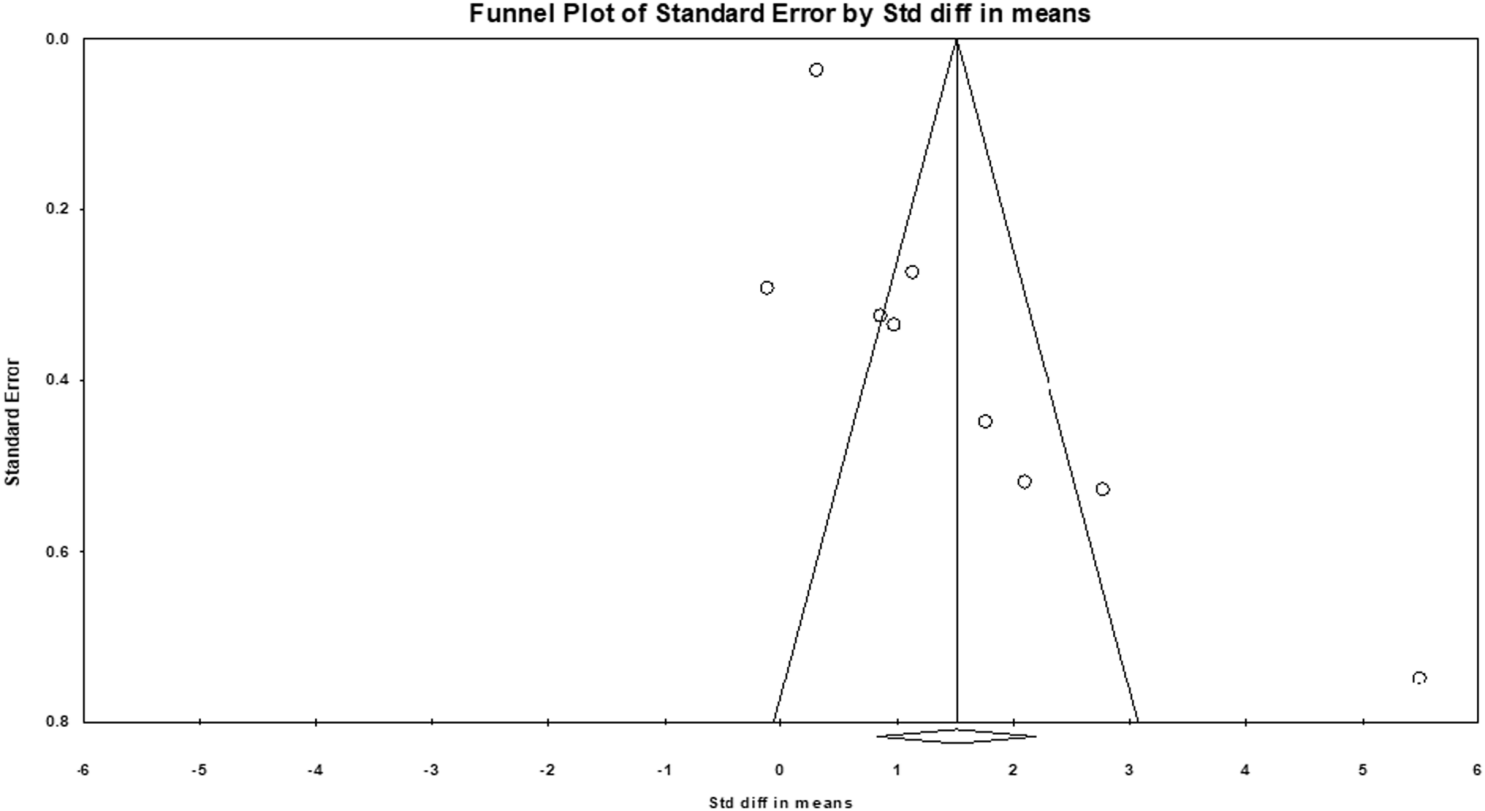
Funnel plot of studies that included speech-in-noise perception scores interpreted using SNR50

Since the overall studies had a variety of interpretations of speech in noise tests, further meta-analyses were divided into three categories based on the way the speech in the noise perception test was scored: (a) SNR50, (b) SNR loss and (c) raw mean scores. The meta-analysis of eight studies with an SNR50 comprising 1045 individuals with tinnitus and 3532 controls yielded a significant effect on speech performance in noise. Using the random-effects model, speech-in-noise perception scores among individuals with tinnitus had a standard mean difference of 2.85 (95% CI 1.68–4.02, p < 0.01) (Fig. 3). The figure depicts the difference in means, standard errors and variance of individual studies, along with the forest plot representation of the same studies. The relative weight (%) represents the sample size. Furthermore, Egger’s regression test, which is the relationship between the effect size estimates and their precision (standard error), showed p = 0.11, which indicated no publication bias. The same funnel plot is depicted in Fig. 4.

**Fig. 5:**
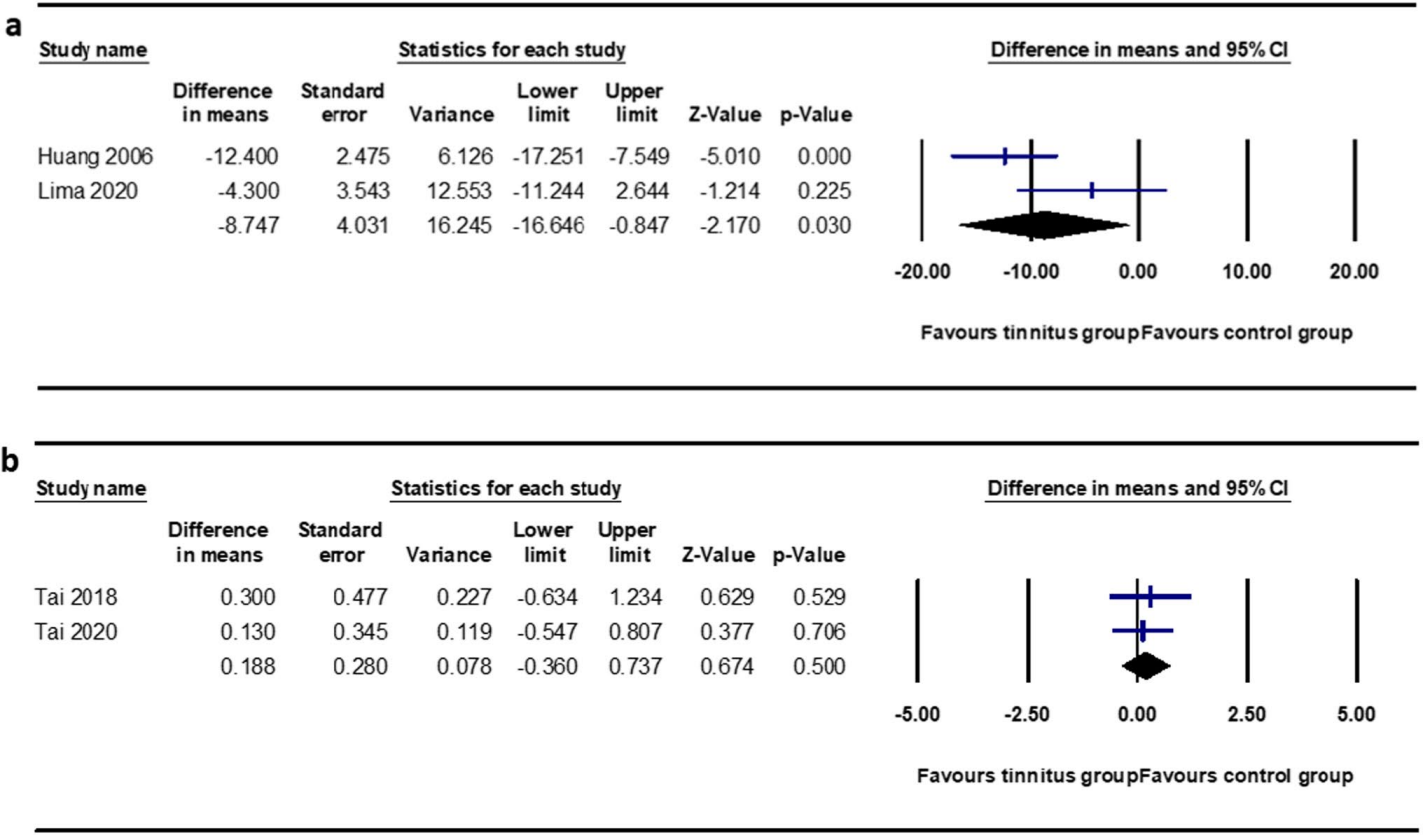
Meta-analysis of speech perception in the noise test interpreted as raw mean scores (above) and SNR loss (below). A total of four studies are computed and depicted along with the respective forest plots

In contrast, the results of the meta-analysis of studies using SNR loss and raw scores as outcome measures were less pronounced. For the raw mean score values, two studies were included in the meta-analysis [16, 20], which indicated a significant mean difference of − 8.747 (95% CI − 16.646 to − 0.847, *p* = 0.03) (Fig. 5a). Similarly, for SNR loss, two studies were included in the meta-analysis [17, 61], and the mean difference was 0.188 (95% CI − 0.360 to 0.737, *p* = 0.50), indicating no significant effect of speech on the noise perception score (Fig. 5b).

Overall, the meta-analysis results suggest that SNR50 scores may be poorer in those with tinnitus than in a control group without tinnitus. Speech in noise perception tests that calculate an SNR loss or a raw percent correct score may be less sensitive to these group differences.

#### Meta-regression

It is well known that factors such as age, hearing acuity and target type affect speech perception in noise scores in addition to tinnitus alone. Therefore, a meta-regression was conducted to investigate the potential role of moderators in speech perception and noise performance.

The covariates used for meta-regression were hearing acuity, age and target-masker type. Q statistics were used to analyze the fit of moderators in one model. Neither the combined model including hearing acuity, age and target type (i.e., intercept) was significant (1.572 ± 1.815; 95% CI − 1.986 to 5.130, p = 0.386), nor did it individually show a significant effect on speech in noise performance (hearing thresholds: − 0.005 ± 0.019, 95% CI − 0.032 to 0.044, p = 0.760; age: − 0.0008 ± 0.041, 95% CI − 0.082 to 0.081, p = 0.985; target type: − 0.786 ± 1.784, 95% CI − 4.283 to 2.711, p = 0.659) (Table 6). This finding implies that hearing acuity and age do not significantly influence speech-in-noise perception scores in tinnitus patients. Based on the goodness of fit, the heterogeneity was high, with significant differences among the moderators (Tau^2^ = 1.739, I^2^ = 93.04%, p = 0.000, R^2^ = 0.00).

**Table 6:** Meta-regression random-effects model: test of the model using three moderators (hearing thresholds, age and target type)

Covariates	Coefficient	Standard error	95% lower	95% upper	Z value	*p *value
Intercept	0.866	0.570	− 0.251	1.984	0.87	0.386
Hearing thresholds	0.005	0.019	− 0.032	0.044	0.30	0.766
Target type	− 0.786	1.784	− 4.283	2.711	− 0.44	0.659
Age	− 0.008	0.041	− 0.032	0.081	− 0.02	0.985
Statistics for Model 1						
Test of the model: Simultaneous test that all coefficients are (excluding intercept) zero			Q = 0.37, df = 3, *p* = 0.94			
Goodness of fit: Test that unexplained variance is zero			Tau^2^ = 1.739, Tau = 1.31, I^2^ = 93.04%, Q = 71.85, df = 5, *p* = 0.0000			
The proportion of total between-study variance explained by Model 1			R^2^ = 0.00 (computed value is − 1.02)			

**Fig. 6:**
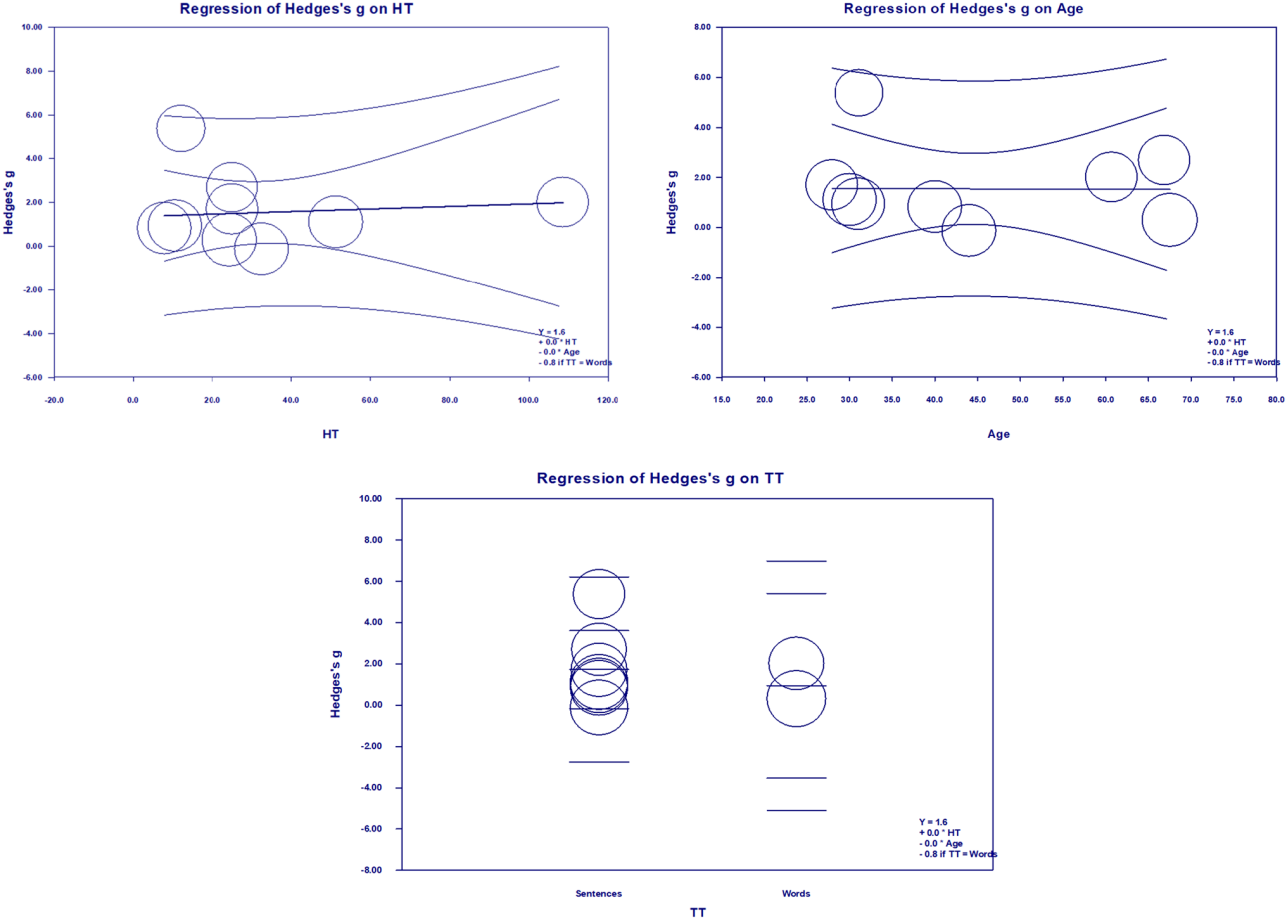
The scatterplot includes a regression line (intercept) and confidence intervals under two conditions: (upper left) depiction based on hearing thresholds (HT), (upper right) depiction of age changes, and (lower) changes in target type. *HT* hearing thresholds, *TT* target type

The results are further depicted using a scatterplot visualizing the meta-regression results of the effect size (Hedges's g) on hearing threholds (Fig. 6a), age (Fig. 6b) and taregt type (Fig. 6c).

## Discussion

Speech perception in noise tests is among the complex auditory tasks and plays a vital role in understanding everyday listening situations. A fair number of studies have explored speech-in-noise measures in tinnitus populations. This review aimed to understand the effect of continuous subjective tinnitus on speech perception in noise scores in general and concerning certain factors.

### The overall impact of tinnitus on speech perception in noise

The overall meta-analysis results suggest that continuous tinnitus significantly negatively impacts speech-in-noise performance. There could be a few possible mechanisms leading to this impact.

*Hearing loss:* First, the primary cause of tinnitus is cochlear damage. This reduces audibility, causing a broadly tuned response of the basilar membrane and negatively affecting temporal resolution and frequency selectivity [63, 64].

Another major cause is the presence of hidden hearing loss. SNHL, especially in aging ears, is often accompanied by tinnitus and hyperacusis. It has been suggested that loss of sensory outflow from the auditory periphery causes a compensatory increase in “central gain”, which underlies these perceptual anomalies, such as tinnitus [65]. In the current review, studies have highlighted that although normal audiometric thresholds exist, increased hearing thresholds at high frequencies (> 2000 Hz) imply damage to inner hair cells or cochlear nerve terminals. The literature reports that acoustic overexposure can lead to rapid, irreversible loss of cochlear nerve terminals, eventually causing slow degeneration of spiral ganglion cells despite the full recovery of thresholds and no loss of OHCs [45].

*Central masking:* The speech in the noise test is generally attributed to a top-down phenomenon. Hence, another major factor leading to poor speech in noise performance is the involvement of the central auditory system (CAS) rather than the periphery. The literature reports that tinnitus affects the primary system as a "central masker" to interrupt speech in noise performance [58]. It has been reported that tinnitus occurs depending on the different plastic changes in the CAS [45].

*Auditory segregation*: The speech-in-noise test requires an individual to segregate speech in the presence of noise. The current literature shows that individuals with tinnitus are less able to use segregation cues regarding talker or masker differences [56]. The segregation ability better represents the release from informational masking and the role of central processing difficulties.

*Cognitive deficits:* Previous literature has shown that tinnitus affects understanding speech in noise due to deficiencies in central processing and/or attention or working memory [28, 66]. Tinnitus affects cognitive abilities in a way that drives cognitive resources away from auditory inputs, making speech-in-noise performance difficult [67]. Previous literature endorses this through details of cognitive tests performed using both behavioral and electrophysiological tests [68]. It is highlighted in the literature that cognitive spare capacity (short-term storage and information processing) tends to decrease for higher-level speech processing in adverse listening conditions [69, 70]. Furthermore, for individuals with tinnitus and normal hearing, this is explained using the information-degradation hypothesis, where tinnitus, as a distracting sound, makes it more effective for speech-in-noise recognition, which results in the depletion of cognitive resources. The current literature associates the impact of poor speech-in-noise performance with auditory attention (mainly selective attention) [4, 17, 20].

In the overall review, the study by Zeng et al. [12] showed no significant difference in speech-in-noise performance between tinnitus patients and controls, even when hearing loss was controlled for. The authors suggested that tinnitus does not interfere with speech-to-noise performance, mainly due to spectral differences. The tinnitus individuals had more difficulty perceiving female talkers than male talkers owing to the high-pitch nature of tinnitus. Similarly, Bures et al. [15] did not observe a significant score change for individuals with tinnitus or individuals in the control group. The authors reported that understanding of speech is more affected by age than tinnitus. If both tinnitus and aging coincide, the effect of age is greater and may mask the effect of tinnitus. However, as the studies did not find a significant difference in speech-to-noise perception scores, they suggest a trend toward poor speech perception in noise tests among individuals with tinnitus.

An overall review and subgroup analysis were performed based on hearing acuity and age to understand various moderating factors for the major impact of speech-in-noise performance in individuals with tinnitus.

### Age and speech perception in noise

Age is a critical factor that independently influences speech perception in noise [54]. The aging process leads to changes in the auditory system and increases vulnerability to noise in the background [71]. Most studies in the current review included individuals with tinnitus (approximately 18–75 years) in a more comprehensive age range. According to the meta-regression analysis, age may not play a crucial role in speech and noise perception in tinnitus patients. Previous studies have suggested that the effect of age on older adults could mask the effect of tinnitus, assuming that the influence of tinnitus on speech perception in noise could be smaller than the effect of age on older adults [15]. However, the current subgroup meta-regression results suggest the opposite, where the addition of age as a factor did not significantly alter speech perception in noise. Since no experimental evidence systematically controls for the effect of age at present, it would be interesting to explore this issue in future studies.

### Hearing acuity and speech in noise perception

It is well known that hearing loss is the most common and independent factor that influences speech perception in noise [72, 73]. Reduced hearing is mainly due to damage to peripheral hearing structures, causing difficulty in discriminating incoming sounds to comprehend speech in noise [74, 75]. In the current review, many studies categorized tinnitus participants based on hearing acuity according to their audiometric thresholds. Hence, a subgroup meta-regression was performed to determine the effect of age. Based on the results, hearing ability may not be the main factor contributing to difficulty understanding speech in noisy environments. Since individuals with tinnitus show poor speech perception in noise scores irrespective of hearing loss, it is possible that tinnitus has a mechanism in higher centers that can result in poor performance concerning speech in noisy environments. This may be due to a central cause, such as a disorder in auditory processing or top-down cognitive deficits.

The literature also reports the inclusion of high-frequency audiometry to determine the influence of speech perception on noise performance. The authors reported that individuals with tinnitus and hearing loss (based on increased high-frequency thresholds) had significantly poorer speech perception based on noise scores [23]. This finding suggested that there might be a different mechanism underlying the pathophysiological mechanism of tinnitus and associated hearing loss. Since there are few studies controlling for high-frequency hearing loss at present, future studies can explore these factors in combination with tests assessing higher centers.

### Speech-in-noise test: measurement and interpretation

The speech-in-noise test is a complex auditory test used to evaluate an individual's ability to understand speech in a noisy background. The speech-in-noise perception test included various target and masker combinations for the real-world simulation. In the current review, most studies used sentences as the target and speech-shaped noise (n = 12) and multitalker babble (n = 11). Sentences are a highly ecologically valid form of stimulus, as they mimic daily communication. They also present various contextual and linguistic cues to make the speech-in-noise perception test more sensitive for assessment. It has been reported that sentences combined with noise for an assessment can better capture the perceptual demands of everyday communication [76]. Furthermore, noise types such as speech-shaped noise and multitalker babble similarly mimic real-world conditions and create informational masking [77].

In the literature, three methods were used for speech in noise perception assessment: SNR 50, SNR loss and raw speech in noise perception scores. The current review consists of most studies skewed toward the SNR 50 as an outcome measure for assessing speech in noise perception and shows a significant negative impact of speech-in-noise scores. SNR50 represents the characteristics of the psychometric function at the SNR level that allows a subject to correctly identify 50% of the signal, i.e., the 50% speech reception threshold (SRT50) and the function's slope [78]. This detailed interpretation of speech in the noise perception test increases the sensitivity and reliability of the results.

The other methods used in the literature are SNR loss and raw score loss. Two studies each reported SNR loss and raw scores [16, 17, 20, 61]. The SNR loss represents the difference in the signal-to-noise ratio between two optimal conditions (with one reference value) in noise, quantifying the degree to which speech perception in noise is affected, and the raw scores are the overall average of the scores per item in the test. SNR loss resulted in no significant impact of speech on noise performance. This may be due to the limited number of studies included in the meta-analysis.

While the SNR loss and SNR 50 are valuable metrics, they differ in a few ways. SNR loss provides a global view of an individual's speech perception affected by noise, allowing for performance comparisons across norms. The SNR50, on the other hand, provides a specific reference point where speech intelligibility is affected for at least 50% of the responses, making it a critical SNR. It also considers parameters such as the presentation level of the target, the number of correct responses, and the total number of items tested per level, ensuring the accuracy of the test. Based on the above variations, the overall significant negative impact of speech perception on noise performance may be most suitable when interpreted using the SNR50.

### Tinnitus characteristics and speech perception in noise

Based on the literature, most of the studies used the THI to assess the severity and impact of tinnitus, followed by the TFI, TRQ and TQ. In the literature, the THI is the most common tool used to assess tinnitus severity. This could be due to the development of the assessment tool much earlier than other tools and the ease of administration, as only three response options are included for each question. However, the literature suggests that the THI questionnaire has not been prospectively evaluated for assessing responsiveness and lacks a comparison of the effectiveness of the outcome [79].

The other commonly used tool in the literature is the TFI (n = 6), the most recently developed tool [48]. It is a tool used to measure the severity and impact of tinnitus and is based on the WHO International Classification of Functioning, Disability and Health (FCF) framework. Consisting of eight subscales, the TFI comprehensively assesses multiple dimensions of tinnitus-related impact. Although the current review consists of a few articles using the TFI, despite its recent development, future studies can include the TFI to measure tinnitus severity and impact to evaluate individuals with tinnitus and group them according to specific problems based on the subscales. This interpretation will have a substantial impact on both assessment and treatment measures.

Tinnitus severity is a fundamental aspect of tinnitus evaluation and provides valuable insights into the subjective perception of the loudness and annoyance of tinnitus. The current literature includes most studies of individuals with minimal to mild tinnitus severity (starting from a score of 15 based on the THI). The literature suggests a lack of correlation between tinnitus severity and speech in noise perception [15, 61]. However, there is no definite hypothesis to explain the lack of correlation except for the heterogeneity of the study sample.

## Conclusion

The main limitation of the current review is the heterogeneity of the tinnitus population. It is challenging to homogenize the groups of participants based on various factors, such as etiology, comorbidities, and psychological factors. Furthermore, the lower sample size in various studies makes it challenging to generalize the findings. Despite these limitations, the current review integrates the findings of each study, addressing mainly the influence of tinnitus on speech in noise perception and other parameters.

According to the current review, tinnitus has an overall effect on speech-in-noise performance, and multiple reasons (causes) are hypothesized for this phenomenon. The literature reports multiple possibilities and hypotheses to account for poor speech recognition scores in noise, but most studies have not proven this. Future studies should explore the correlation between reduced speech perception in noise scores and other factors that may influence this correlation.

## Data Availability

All the necessary data has been provided.

## References

[1] Bhatt JM, Lin HW, Bhattacharyya N (2016) Prevalence, severity, exposures, and treatment patterns of tinnitus in the United States. JAMA Otolaryngol Neck Surg 142(10):959. 10.1001/jamaoto.2016.1700 10.1001/jamaoto.2016.1700 PMC5812683 27441392

[2] De Ridder D, Schlee W, Vanneste S, Londero A, Weisz N, Kleinjung T, Shekhawat GS, Elgoyhen AB, Song J-J, Andersson G, Adhia D, de Azevedo AA, Baguley DM, Biesinger E, Binetti AC, Del Bo L, Cederroth CR, Cima R, Eggermont JJ, Figueiredo R, Fuller TE, Gallus S, Gilles A, Hall DA, Van de Heyning P, Hoare DJ, Khedr EM, Kikidis D, Kleinstaeuber M, Kreuzer PM, Lai J-T, Lainez JM, Landgrebe M, Li LP-H, Lim HH, Liu T-C, Lopez-Escamez JA, Mazurek B, Moller AR, Neff P, Pantev C, Park SN, Piccirillo JF, Poeppl TB, Rauschecker JP, Salvi R, Sanchez TG, Schecklmann M, Schiller A, Searchfield GD, Tyler R, Vielsmeier V, Vlaeyen JWS, Zhang J, Zheng Y, de Nora M, Langguth B (2021) Tinnitus and tinnitus disorder: theoretical and operational definitions (an international multidisciplinary proposal). Elsevier, Amsterdam 10.1016/bs.pbr.2020.12.002 33637213

[3] Gallo KEB et al (2023) Effect of tinnitus on sleep quality and insomnia. Int Arch Otorhinolaryngol 27(02):e197–e202. 10.1055/s-0041-1735455 37125358 10.1055/s-0041-1735455 PMC10147471

[4] Hennig TR, Costa MJ, Urnau D, Becker KT, Schuster LC (2011) Recognition of speech of normal-hearing individuals with tinnitus and hyperacusis. Int Arch Otorhinolaryngol 15(1):21–28

[5] Makar SK, Biswas A, Shatapathy P (2014) The impact of tinnitus on sufferers in Indian population. Indian J Otolaryngol Head Neck Surg 66(SUPPL. 1):37–51. 10.1007/s12070-011-0291-x 24533358 10.1007/s12070-011-0291-x PMC3918304

[6] Han BI, Lee HW, Kim TY, Lim JS, Shin KS (2009) Tinnitus: characteristics, causes, mechanisms, and treatments. J Clin Neurol Korea 5(1):11–19. 10.3988/jcn.2009.5.1.11 10.3988/jcn.2009.5.1.11 PMC2686891 19513328

[7] Heinrich A (2021) The role of cognition for speech-in-noise perception: considering individual listening strategies related to aging and hearing loss. Int J Behav Dev 45(5):382–388. 10.1177/0165025420914984

[8] Lad M, Holmes E, Chu A, Griffiths TD (2020) Speech-in-noise detection is related to auditory working memory precision for frequency. Sci Rep 10(1):13997. 10.1038/s41598-020-70952-9 32814792 10.1038/s41598-020-70952-9 PMC7438331

[9] Schoof T, Rosen S (2014) The role of auditory and cognitive factors in understanding speech in noise by normal-hearing older listeners. Front Aging Neurosci. 10.3389/fnagi.2014.00307 25429266 10.3389/fnagi.2014.00307 PMC4228854

[10] Guest H, Munro KJ, Plack CJ (2019) Acoustic middle-ear-muscle-reflex thresholds in humans with normal audiograms: no relations to tinnitus, speech perception in noise, or noise exposure. Neuroscience 407:75–82. 10.1016/j.neuroscience.2018.12.019 30579832 10.1016/j.neuroscience.2018.12.019

[11] Minami SB, Oishi N, Watabe T, Uno K, Ogawa K (2018) Auditory related resting state fMRI functional connectivity in tinnitus patients: tinnitus diagnosis performance. Otol Neurotol 39(1):1–5. 10.1097/MAO.0000000000001626 29210942 10.1097/MAO.0000000000001626

[12] Zeng FG, Richardson M, Turner K (2020) Tinnitus does not interfere with auditory and speech perception. J Neurosci 40(31):6007–6017. 10.1523/JNEUROSCI.0396-20.2020 32554549 10.1523/JNEUROSCI.0396-20.2020 PMC7392513

[13] Araneda R et al (2015) Altered top-down cognitive control and auditory processing in tinnitus: evidences from auditory and visual spatial stroop. Restor Neurol Neurosci 33(1):67–80. 10.3233/RNN-140433 25420904 10.3233/RNN-140433

[14] Heeren A et al (2014) Tinnitus specifically alters the top-down executive control sub-component of attention: evidence from the Attention Network Task. Behav Brain Res 269:147–154. 10.1016/j.bbr.2014.04.043 24793493 10.1016/j.bbr.2014.04.043

[15] Bureš Z, Profant O, Svobodová V, Tóthová D, Vencovský V, Syka J (2019) Speech comprehension and its relation to other auditory parameters in elderly patients with tinnitus. Front Aging Neurosci 11:1–12. 10.3389/fnagi.2019.00219 31496946 10.3389/fnagi.2019.00219 PMC6713070

[16] Huang CY, Lee HH, Chung KC, Chen HC, Shen YJ, Wu JL (2006) Relationships among speech perception, self-rated tinnitus loudness and disability in tinnitus patients with normal pure-tone thresholds of hearing. Orl 69(1):25–29. 10.1159/000096713 17085949 10.1159/000096713

[17] Tai Y, Husain FT (2020) Association between tinnitus pitch and consonant recognition in noise. Am J Audiol 29(4):916–929. 10.1044/2020_AJA-20-00050 33237797 10.1044/2020_AJA-20-00050

[18] Jain C, Sahoo JP (2014) The effect of tinnitus on some psychoacoustical abilities in individuals with normal hearing sensitivity. Int Tinnitus J 19(1):28–35. 10.5935/0946-5448.20140004 27186830 10.5935/0946-5448.20140004

[19] Jensen JJ, Callaway SL, Lunner T, Wendt D (2018) Measuring the impact of tinnitus on aided listening effort using pupillary response. Trends Hear 22:1–17. 10.1177/2331216518795340 10.1177/2331216518795340 PMC6136111 30205768

[20] Lima DO, de Araújo AMGD, Branco-Barreiro FCA, da Carneiro CS, Almeida LNA, da Rosa MRD (2020) Auditory attention in individuals with tinnitus. Braz J Otorhinolaryngol 86(4):461–467. 10.1016/j.bjorl.2019.01.011 30926455 10.1016/j.bjorl.2019.01.011 PMC9422633

[21] Manning C, Mermagen T, Scharine A (2017) The effect of sensorineural hearing loss and tinnitus on speech recognition over air and bone conduction military communications headsets. Hear Res 349:67–75. 10.1016/j.heares.2016.10.019 27989949 10.1016/j.heares.2016.10.019

[22] Mondelli MFCG, Cabreira AF, Matos ILD, Ferreira MC, Rocha AV (2021) Sound generator: analysis of the effectiveness of noise in the habituation of tinnitus. Int Arch Otorhinolaryngol 25(2):205–212. 10.1055/s-0040-1713377 10.1055/s-0040-1713377 PMC8096505 33968221

[23] Oosterloo BC, Homans NC, Goedegebure A (2020) Tinnitus affects speech in noise comprehension in individuals with hearing loss. Otol Neurotol 41(9):E1074–E1081. 10.1097/MAO.0000000000002733 32925836 10.1097/MAO.0000000000002733

[24] Ryu IS, Ahn JH, Lim HW, Joo KY, Chung JW (2012) Evaluation of masking effects on speech perception in patients with unilateral chronic tinnitus using the hearing in noise test. Otol Neurotol 33(9):1472–1476. 10.1097/MAO.0b013e31826dbcc4 22996163 10.1097/MAO.0b013e31826dbcc4

[25] Kohansal B, Asghari M, Najafi S, Hamedi F (2021) Effect of tinnitus on the performance of central auditory system: a review. Audit Vestib Res. 10.18502/avr.v30i4.7444

[26] Lee H-J, Shim YJ, Chang M (2023) Tinnitus and cognitive decline. Korean J Otorhinolaryngol Head Neck Surg 66(10):641–645. 10.3342/kjorl-hns.2023.00913

[27] Ruan Q, Chen B, Panza F (2023) Which came first, age-related hearing loss with tinnitus or cognitive impairment? What are the potential pathways? J Integr Neurosci 22(5):109. 10.31083/j.jin2205109 37735130 10.31083/j.jin2205109

[28] Ivansic D, Guntinas-Lichius O, Müller B, Volk GF, Schneider G, Dobel C (2017) Impairments of speech comprehension in patients with tinnitus—a review. Front Aging Neurosci. 10.3389/fnagi.2017.00224 28744214 10.3389/fnagi.2017.00224 PMC5504093

[29] Pichora-Fuller MK, Souza PE (2003) Effects of aging on auditory processing of speech. Int J Audiol 42(sup2):11–16. 10.3109/14992020309074638 12918623

[30] Babkoff H, Fostick L (2017) Age-related changes in auditory processing and speech perception: cross-sectional and longitudinal analyses. Eur J Ageing 14(3):269–281. 10.1007/s10433-017-0410-y 28936137 10.1007/s10433-017-0410-y PMC5587455

[31] Buss E, Calandruccio L, Oleson J, Leibold LJ (2021) Contribution of stimulus variability to word recognition in noise versus two-talker speech for school-age children and adults. Ear Hear 42(2):313–322. 10.1097/AUD.0000000000000951 32881723 10.1097/AUD.0000000000000951 PMC7897187

[32] Summers WV, Pisoni DB, Bernacki RH, Pedlow RI, Stokes MA (1988) Effects of noise on speech production: acoustic and perceptual analyses. J Acoust Soc Am 84(3):917–928. 10.1121/1.396660 3183209 10.1121/1.396660 PMC3507387

[33] Hunter CR, Pisoni DB (2018) Extrinsic cognitive load impairs spoken word recognition in high- and low-predictability sentences. Ear Hear 39(2):378–389. 10.1097/AUD.0000000000000493 28945658 10.1097/AUD.0000000000000493 PMC5821552

[34] Iyer N, Brungart DS, Simpson BD (2010) Effects of target-masker contextual similarity on the multimasker penalty in a three-talker diotic listening task. J Acoust Soc Am 128(5):2998–3010. 10.1121/1.3479547 21110595 10.1121/1.3479547

[35] Page MJ et al (2021) The PRISMA 2020 statement: an updated guideline for reporting systematic reviews. BMJ. 10.1136/bmj.n71 33782057 10.1136/bmj.n71 PMC8005924

[36] Higgins JPT, Thomas J, Chandler J, Cumpston M, Li T, Page MJ, Welch VA (eds) (2023) Cochrane handbook for systematic reviews of interventions version 6.4. Cochrane [Online]. www.training.cochrane.org/handbook. Accessed Sept 2023

[37] Rohatgi A (2022) “WebPlotDigitizer.” Pacifica, CA, USA. [Online]. https://automeris.io/WebPlotDigitizer. Accessed June 2023

[38] “Critical Appraisal Skills Programme CASP Checklists.” 2013. [online]. https://casp-uk.net/#!checklists/cb36. Accessed Mar 2023

[39] Borenstein M, Hedges L, Higgins J, Rothstein H (2022) Comprehensive meta-analysis. Biostat, Englewood

[40] Borenstein M, Hedges LV, Higgins JPT, Rothstein HR (2010) A basic introduction to fixed-effect and random-effects models for meta-analysis. Res Synth Methods 1(2):97–111. 10.1002/jrsm.12 26061376 10.1002/jrsm.12

[41] Shim HJ, Cho YT, Kim DH, Choi JH (2021) Influence of tinnitus on auditory spectral and temporal resolution and speech perception in tinnitus patients. J Neurosci 35(42):14260–14269. 10.1523/JNEUROSCI.5091-14.2015 10.1523/JNEUROSCI.5091-14.2015 PMC6605422 26490865

[42] Cheng LH, Wang CH, Lu RH, Chen YF (2020) Evaluating the function of the medial olivocochlear bundle in patients with bilateral tinnitus. J Speech Lang Hear Res 63(6):1969–1978. 10.1044/2020_JSLHR-19-00080 32511051 10.1044/2020_JSLHR-19-00080

[43] Degeest S, Kestens K, Keppler H (2022) Investigation of the relation between tinnitus, cognition, and the amount of listening effort. J Speech Lang Hear Res 65(5):1988–2002. 10.1044/2022_JSLHR-21-00347 35377707 10.1044/2022_JSLHR-21-00347

[44] Eynde CV, Denys S, Desloovere C, Wouters J, Verhaert N (2016) Speech-in-noise testing as a marker for noise-induced hearing loss and tinnitus. B-ENT 1:185–191 29461742

[45] Gilles A, Schlee W, Rabau S, Wouters K, Fransen E, de Heyning PV (2016) Decreased speech-in-noise understanding in young adults with tinnitus. Front Neurosci 10:1–14. 10.3389/fnins.2016.00288 27445661 10.3389/fnins.2016.00288 PMC4923253

[46] Jagoda L, Giroud N, Neff P, Kegel A, Kleinjung T, Meyer M (2018) Speech perception in tinnitus is related to individual distress level—a neurophysiological study. Hear Res 367:48–58. 10.1016/j.heares.2018.07.001 30031353 10.1016/j.heares.2018.07.001

[47] Newman CW, Jacobson GP, Spitzer JB (1996) Development of the Tinnitus Handicap Inventory. Arch Otolaryngol Head Neck Surg 122(2):143–148. 10.1001/archotol.1996.01890140029007 8630207 10.1001/archotol.1996.01890140029007

[48] Meikle MB et al (2012) Erratum: The tinnitus functional index: Development of a new clinical measure for chronic, intrusive tinnitus (Ear and Hearing (2012) 33 (153–176)). Ear Hear 33(3):443. 10.1097/AUD.0b013e3182597b3e 10.1097/AUD.0b013e31822f67c0 22156949

[49] Hallam RS, Jakes SC, Hinchcliffe R (1988) Cognitive variables in tinnitus annoyance. Br J Clin Psychol 27(3):213–222. 10.1111/j.2044-8260.1988.tb00778.x 3191301 10.1111/j.2044-8260.1988.tb00778.x

[50] Wilson PH, Henry J, Bowen M, Haralambous G (1991) Tinnitus reaction questionnaire: psychometric properties of a measure of distress associated with tinnitus. J Speech Hear Res 34(1):197–201 2008074

[51] Arts RAGJ, George ELJ, Janssen MAML, Griessner A, Zierhofer C, Stokroos RJ (2018) The effect of tinnitus specific intracochlear stimulation on speech perception in patients with unilateral or asymmetric hearing loss accompanied with tinnitus and the effect of formal auditory training. Int J Audiol 57(6):426–439. 10.1080/14992027.2017.1408964 29188740 10.1080/14992027.2017.1408964

[52] Lindblad AC, Rosenhall U, Olofsson Å, Hagerman B (2014) Tinnitus and other auditory problems—occupational noise exposure below risk limits may cause inner ear dysfunction. PLoS ONE. 10.1371/journal.pone.0097377 24827149 10.1371/journal.pone.0097377 PMC4020865

[53] Mertens G, Punte AK, Bodt MD, Heyning PVD (2015) Binaural auditory outcomes in patients with postlingual profound unilateral hearing loss: 3 years after cochlear implantation. Audiol Neurotol 20(suppl 1):67–72. 10.1159/000380751 10.1159/000380751 25997790

[54] Vermeire K, Heyning PVD (2009) Binaural hearing after cochlear implantation in subjects with unilateral sensorineural deafness and tinnitus. Audiol Neurotol 14(3):163–171. 10.1159/000171478 10.1159/000171478 19005250

[55] Newman CW, Wharton JA, Shivapuja BG, Jacobson GP (1994) Relationships among psychoacoustic judgments, speech understanding ability and self-perceived handicap in tinnitus subjects. Int J Audiol 33(1):47–60. 10.3109/00206099409072954 10.3109/00206099409072954 8129680

[56] Liu YW, Wang B, Chen B, Galvin JJ, Fu QJ (2020) Tinnitus impairs segregation of competing speech in normal-hearing listeners. Sci Rep 10(1):1–11. 10.1038/s41598-020-76942-1 33199782 10.1038/s41598-020-76942-1 PMC7670434

[57] Neff P et al (2021) The impact of tinnitus distress on cognition. Sci Rep 11(1):1–9. 10.1038/s41598-021-81728-0 33500489 10.1038/s41598-021-81728-0 PMC7838303

[58] Moon IJ, Won JH, Kang HW, Kim DH, An Y-H, Shim HJ (2015) Influence of tinnitus on auditory spectral and temporal resolution and speech perception in tinnitus patients. J Neurosci 35(42):14260–14269. 10.1523/JNEUROSCI.5091-14.2015 26490865 10.1523/JNEUROSCI.5091-14.2015 PMC6605422

[59] Parthasarathy S, Shetty HN (2021) Manipulation of hearing aid gain and tinnitus relief: a paired comparison study. J Int Adv Otol 17(2):145–149. 10.5152/JIAO.2021.8873 33893784 10.5152/JIAO.2021.8873 PMC9449996

[60] Henry JA et al (2017) Tinnitus management: randomized controlled trial comparing extended-wear hearing AIDS, conventional hearing AIDS, and combination instruments. J Am Acad Audiol 28(6):546–561. 10.3766/jaaa.16067 28590898 10.3766/jaaa.16067

[61] Tai Y, Husain FT (2018) Right-ear advantage for speech-in-noise recognition in patients with nonlateralized tinnitus and normal hearing sensitivity. JARO J Assoc Res Otolaryngol 19(2):211–221. 10.1007/s10162-017-0647-3 29181615 10.1007/s10162-017-0647-3 PMC5878148

[62] Távora-Vieira D, Marino R, Acharya A, Rajan GP (2015) The impact of cochlear implantation on speech understanding, subjective hearing performance, and tinnitus perception in patients with unilateral severe to profound hearing loss. Otol Neurotol 36(3):430–436. 10.1097/MAO.0000000000000707 25594387 10.1097/MAO.0000000000000707

[63] Glasberg BR, Moore BCJ (1986) Auditory filter shapes in subjects with unilateral and bilateral cochlear impairments. J Acoust Soc Am 79(4):1020–1033. 10.1121/1.393374 3700857 10.1121/1.393374

[64] Oxenham AJ, Bacon SP (2003) Cochlear compression: perceptual measures and implications for normal and impaired hearing. Ear Hear 24(5):352–366. 10.1097/01.AUD.0000090470.73934.78 14534407 10.1097/01.AUD.0000090470.73934.78

[65] Liberman MC, Epstein MJ, Cleveland SS, Wang H, Maison SF (2016) Toward a differential diagnosis of hidden hearing loss in humans. PLoS ONE 11(9):e0162726. 10.1371/journal.pone.0162726 27618300 10.1371/journal.pone.0162726 PMC5019483

[66] Rossiter S, Stevens C, Walker G (2006) Tinnitus and its effect on working memory and attention. J Speech Lang Hear Res 49(1):150–160. 10.1044/1092-4388(2006/012) 16533080 10.1044/1092-4388(2006/012)

[67] Tegg-Quinn S, Bennett RJ, Eikelboom RH, Baguley DM (2016) The impact of tinnitus upon cognition in adults: a systematic review. Int J Audiol 55(10):533–540. 10.1080/14992027.2016.1185168 27240696 10.1080/14992027.2016.1185168

[68] Vasudevan H, Ganapathy K, Palaniswamy HP, Searchfield G, Rajashekhar B (2021) Systematic review and meta-analysis on the effect of continuous subjective tinnitus on attention and habituation. PeerJ 9:1–25. 10.7717/peerj.12340 10.7717/peerj.12340 PMC8628620 34900408

[69] Rönnberg J, Rudner M, Lunner T (2011) Cognitive hearing science: the legacy of Stuart Gatehouse. Trends Amplif 15(3):140–148. 10.1177/1084713811409762 21606047 10.1177/1084713811409762 PMC4040830

[70] Rudner M, Lunner T (2014) Cognitive spare capacity and speech communication: a narrative overview. BioMed Res Int 2014:869726. 10.1155/2014/869726 24971355 10.1155/2014/869726 PMC4058272

[71] Recanzone G (2018) The effects of aging on auditory cortical function. Hear Res 366:99–105. 10.1016/j.heares.2018.05.013 29853323 10.1016/j.heares.2018.05.013 PMC6103827

[72] Heutink F et al (2021) Factors influencing speech perception in adults with a cochlear implant. Ear Hear 42(4):949–960. 10.1097/AUD.0000000000000988 33480623 10.1097/AUD.0000000000000988 PMC8221708

[73] McCreery RW, Walker EA, Spratford M, Lewis D, Brennan M (2019) Auditory, cognitive, and linguistic factors predict speech recognition in adverse listening conditions for children with hearing loss. Front Neurosci 13:1093. 10.3389/fnins.2019.01093 31680828 10.3389/fnins.2019.01093 PMC6803493

[74] Anderson S, White-Schwoch T, Choi HJ, Kraus N (2013) Training changes processing of speech cues in older adults with hearing loss. Front Syst Neurosci. 10.3389/fnsys.2013.00097 24348347 10.3389/fnsys.2013.00097 PMC3842592

[75] Narne VK (2013) Temporal processing and speech perception in noise by listeners with auditory neuropathy. PLoS ONE 8(2):e55995. 10.1371/journal.pone.0055995 23409105 10.1371/journal.pone.0055995 PMC3567005

[76] Miles KM, Keidser G, Freeston K, Beechey T, Best V, Buchholz JM (2020) Development of the everyday conversational sentences in noise test. J Acoust Soc Am 147(3):1562–1576. 10.1121/10.0000780 32237858 10.1121/10.0000780 PMC7060086

[77] Gundmi A, Himaja P, Dhamani A (2018) Effectiveness of multitalker babble over speech noise and its implications: a comparative study. Indian J Otol 24(2):88. 10.4103/indianjotol.indianjotol_24_18

[78] Reynard P, Lagacé J, Joly C-A, Dodelé L, Veuillet E, Thai-Van H (2022) Speech-in-noise audiometry in adults: a review of the available tests for french speakers. Audiol Neurotol 27(3):185–199. 10.1159/000518968 10.1159/000518968 34937024

[79] Kamalski DM, Hoekstra CE, Zanten BGV, Grolman W, Rovers MM (2010) Measuring disease-specific health-related quality of life to evaluate treatment outcomes in tinnitus patients: a systematic review. Otolaryngol Head Neck Surg 143(2):181–185. 10.1016/j.otohns.2010.03.026 20647116 10.1016/j.otohns.2010.03.026

[80] Liu YW, Cheng X, Chen B, Peng K, Ishiyama A, Fu QJ (2018) Effect of tinnitus and duration of deafness on sound localization and speech recognition in noise in patients with single-sided deafness. Trends Hear. 10.1177/2331216518813802 30509148 10.1177/2331216518813802 PMC6291880

[81] Tugumia D, Samelli AG, Matas CG, Magliaro FCL, Rabelo CM (2016) Auditory training program in subjects with tinnitus. Codas 28(1):27–33. 10.1590/2317-1782/20162015113 27074186 10.1590/2317-1782/20162015113

